# Meta-Analysis of Heterogeneity in the Effects of Wildfire Smoke Exposure on Respiratory Health in North America

**DOI:** 10.3390/ijerph16060960

**Published:** 2019-03-18

**Authors:** Michelle C. Kondo, Anneclaire J. De Roos, Lauren S. White, Warren E. Heilman, Miranda H. Mockrin, Carol Ann Gross-Davis, Igor Burstyn

**Affiliations:** 1 Northern Research Station, USDA Forest Service, Baltimore, MA 21228, USA; mhmockrin@fs.fed.us; 2 School of Public Health, Environmental and Occupational Health, Drexel University, Philadelphia, PA 19104, USA; aderoos@drexel.edu (A.J.D.R.); laurenswhite@gmail.com (L.S.W.); Igor.Burstyn@drexel.edu (I.B.); 3 Northern Research Station—Climate, Fire, and Carbon Cycle Sciences, USDA Forest Service, Lansing, MI 48910, USA; wheilman@fs.fed.us; 4 Office of Air Monitoring & Analysis (3AP40), Air Protection Division, US Environmental Protection Agency, Philadelphia, PA 19103, USA; gross-davis.carolann@epa.gov

**Keywords:** wildfire smoke, respiratory health, heterogeneity of effects, ratio of relative risk

## Abstract

Epidemiological studies consistently show an association between wildfire-related smoke exposure and adverse respiratory health. We conducted a systematic review of evidence in published literature pertaining to heterogeneity of respiratory effects from this exposure in North America. We calculated the within-study ratio of relative risks (RRR) and 95% confidence intervals (CI) to examine heterogeneity of effect by population subgroup, and then summarized the RRRs using meta-analysis. We found evidence of a greater effect of wildfire smoke on respiratory health among females relative to males for asthma (RRR: 1.035, 95% CI: 1.013, 1.057) and chronic obstructive pulmonary disease (RRR: 1.018, 95% CI: 1.003, 1.032). There was evidence of a lower relative risk for all respiratory outcomes among youth compared to adults (RRR: 0.976, 95% CI: 0.963, 0.989). We also found wildfire smoke effects stratified by income, race, education, health behaviors, access to care, housing occupancy, geographic region, and urban/rural status. However, data were insufficient to quantitatively evaluate effect modification by these characteristics. While we found evidence that certain demographic subgroups of the population are more susceptible to respiratory health outcomes from wildfire smoke, it is unclear whether this information can be used to inform policy aimed to reduce health impact of wildfires.

## 1. Introduction

In the Western US and Canada, the annual number of fires, the length of wildfire season, and the total area burned have all increased at a rapid rate over the past 30 years [1,2]. Between 1984 and 2011, the average number of large fires in the US increased by seven per year, and total burn area increased by 355 km^2^ on average per year [2]. Rapidly expanding residential development within and in close proximity to wildland vegetation has the dual outcomes of increasing wildfire ignitions and placing more homes and people at risk of wildfire [3]. Furthermore, climate conditions conducive to more wildfire occurrences (i.e., increased fuel loads, longer fire seasons, larger burn areas, and increased fire emissions) are predicted for the future [4,5,6]. The introduction of and devastation caused by invasive pests, such as the pine beetle, are also influenced by climate change and could influence wildfire area or intensity [7]. 

Impacts of forest fires are wide-ranging, and include emission of particles and gases that degrade air quality [8]. Wildfire smoke contains pollutants, such as carbon monoxide (CO), ultrafine, fine and coarse particulate matter (PM), nitrogen oxides (NOx) and their precursors, polycyclic aromatic hydrocarbons, and volatile organic compounds [9,10]. NOx, CO, PM, and ozone are all regulated under the Clean Air Act and have National Ambient Air Quality Standards in place to protect public health. Among the pollutants from wildfire smoke, as in vehicle emissions, the most consistently present is PM [10]. The chemical composition of wildfire smoke, and the resulting lung toxicity, depends on the landscape, climate, season, burn conditions, available fuels, and phase of combustion (e.g., flaming and smoldering) [11]. Wildfires will produce different chemicals depending on wet or dry conditions of the biomass and higher outputs of PM and other compounds depending on phase of combustion [12].

Particulate matter has been the primary wildfire-related product of interest because of its known relationship to human health. Epidemiological studies consistently show an association between wildfire-related PM exposure and adverse respiratory health outcomes, indicated by hospital and emergency department (ED) admissions, and mortality [13,14,15].

Based on nonwildfire-specific studies of air pollution, susceptibility is associated with preexisting diseases, age, and socioeconomic status [16,17]. Among epidemiological studies that estimate the effects of fine PM on respiratory health at a population level, there is consensus that there is heterogeneity of these associations [18]. Previous reviews have reported inconclusive evidence of heterogeneity of effects of wildfire smoke exposure [15]. However, greater understanding of susceptibility among specific subpopulations to exposure to wildfire smoke could be used to assess and manage health-related impacts of a phenomenon that is common in many areas of the US and may become even more of an issue in the future.

Understanding of susceptibility among specific subpopulations to exposure to wildfire smoke might be used to plan alternative methods to reduce wildfire. For example, there have been calls for more prescribed burning, as opposed to fire suppression, to reduce wildfire smoke impacts on populations [19]. While suppression has been the primary wildfire management strategy in the US, it is possible that public health impacts could be reduced using prescribed burning or other techniques such as reducing surface fuels, forest thinning, or altering forest characteristics like maximum height or density [20,21]. However, techniques that alter temporal or spatial patterns of smoke emissions require assessment of whether effect varies by subpopulation. Understanding of susceptibility among specific subpopulations to exposure to wildfire smoke might also be used to support management that reduces the incidence of large, severe wildfires, or to support appropriate, targeted public health messaging to relatively vulnerable groups. 

We aimed to conduct a review of exposure–response for ED and/or hospital admissions for respiratory conditions in response to air pollution episodes associated with wildfire (including wildfires, forest fires, and peat fires). Our primary goal was to examine heterogeneity of effect between subpopulation groups—specifically, evidence of effect modification by sociodemographic factors. We focused only on respiratory health outcomes among studies conducted in North America to reduce heterogeneity of exposure due to variability in composition of wildfire smoke (most often quantified as particulate matter), dependent on regional climatic and landscape factors, as well as to narrow the health outcome, since any heterogeneity of effect may be outcome-specific.

## 2. Materials and Methods

We identified studies that reported exposure–response for ED and/or hospital admissions for respiratory conditions in response to air pollution episodes associated with wildfire (including wildfires, forest fires, and peat fires) in North America. We followed Preferred Reporting Items for Systematic Reviews and Meta-Analyses (PRISMA) guidelines [22]. We searched biomedical databases in October 2017, including Web of Science, PubMed, and Ovid. 

We submitted a standard Boolean search phrase, with syntax tailored to each database. We submitted the following search phrase to all databases: (wildfire* or forest fire*) and (emergency or hospital* or mortality or asthma* or *respiratory). We excluded studies that

1. Were not related to wildfire exposure.
2. Were not conducted in North America.
3. Were case studies, meta-analyses, editorials, or commentaries.
4. Were not of acute respiratory health outcomes including ED and/or hospital admissions.
5. Did not report estimates of relative risk. We considered odds ratios to be estimates of relative risk (RR) under the rare outcome assumption.

**Figure 1:**
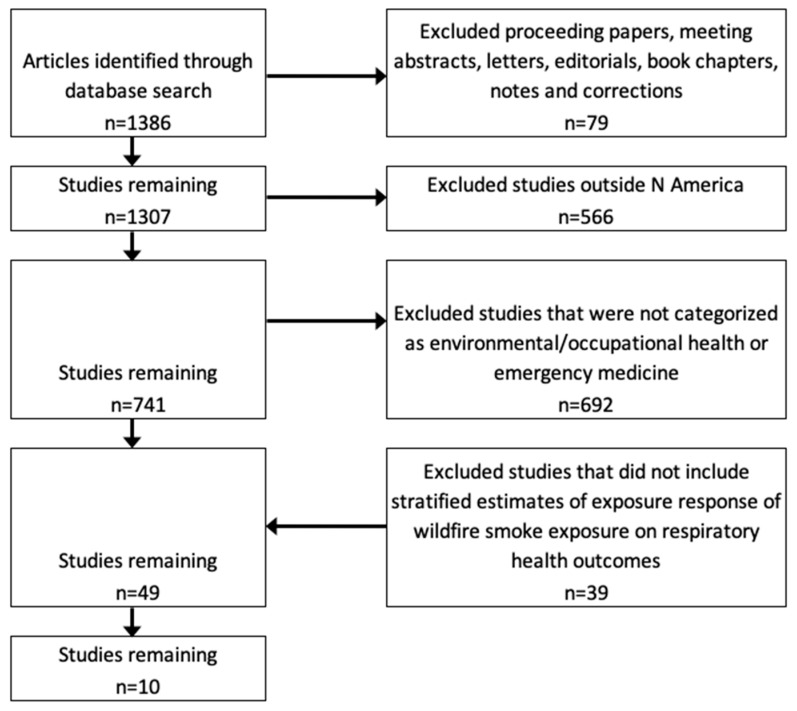
Selection process for studies of wildfire-related particle exposure and respiratory health outcomes.

We did not restrict the timeframe of our literature search. Figure 1 illustrates the study selection process using our search of the Web of Science database, which encompassed all findings from other database searches. The initial searches identified 1386 items. We first excluded studies that were not journal articles (such as commentaries, book chapters or conference proceedings), leaving 1307 reports. We then excluded 566 reports from studies conducted outside of North America, and studies not related to wildfire exposure, leaving 741 studies. Next we excluded 692 studies that were not categorized as environmental or occupational health or emergency medicine topics, leaving 49 studies. Two authors (M.K. and L.W.) then independently reviewed the remaining papers to determine eligibility. We excluded all papers that did not evaluate the association between exposure to wildfire smoke and risk of respiratory health outcomes, with estimation of the relative risk, leaving 10 articles. 

From each study, we recorded research aims, study design, health data source and location, wildfire name and location, study duration, population demographics, wildfire pollution assessment method, exposure contrast for relative risk estimation, lags considered, and method of assessment of health outcomes. 

We documented stratified effect estimates from each study. We obtained effect estimates from three studies [23,24,25] by contacting the authors. In the situation that a study reported multiple effect estimates per stratification group, we included only one estimate from each study using the following prioritization for selection.

1. The shortest available lag time.
2. If multiple estimates were available using different exposure periods (for example, during fire and postfire), we chose estimates associated with exposures during fire (compared to referent period/unit).
3. Where possible, we chose models of combined admission types (both hospital and ED admissions). In the one study that reported separate models on hospital and ED admissions, we chose the outcome with highest number of events, which was ED admissions [26].
4. If multiple predicted exposure definitions were used to produce relative risk estimates (for example, Gan et al. [23]), we chose the exposure definition that provided better reliability according to model fit, of surface measurements of PM_2.5_ (particulate matter of diameter less than 2.5 micrometers).

We wanted to know whether the magnitude of effect differed by different strata (for example, female and male). We cannot statistically assess difference between two RRs, and therefore, for each pair in the stratum (for example, female and male), we used RR estimates to calculate the ratio of relative risks (RRR) [27]. Calculation of the RRRs, and subsequent meta-analysis of RRRs, places confidence limits around the difference in effect between subgroups or strata. 

We translated all RR estimates to the 10 µg/m^3^ contrast prior to calculating RRR. Where possible, we conducted a fixed effects meta-analysis of the RRRs producing a meta-estimate (meta-RRR), and 95% confidence interval for the RRR for each stratum pair. In addition, we report the *I*^2^ statistic which indicates the percent of variation across studies that is due to heterogeneity instead of by chance [28,29]. This statistic does not depend on the number of studies included in the meta-analysis. We implemented all calculations using Stata (v15.1, StataCorp, College Station, TX, USA).

As a sensitivity analysis, we calculated meta-RRR within each category by removing each study in turn. We then compared the resulting meta-RRR and *I*^2^ statistic to check for undue influence of any one study on meta-RRR. 

## 3. Results

### 3.1. Study Characteristics

Table 1 shows characteristics of the 10 studies in our analysis. They were published between 2008 and 2018, and were based on wildfire events that occurred between 2003 and 2012. All studies were based on samples drawn from the US.

Studies utilized a variety of research designs. Six studies used a time series approach [24,26,31,32,34,35], three studies used case crossover design [23,30,32], and two were ecological [25,33].

### 3.2. Smoke Exposure Measurement and Exposure Contrast

Studies used two different approaches to model relative risk associated with wildfire smoke exposure. Eight studies modeled relative risk per unit increase in PM_2.5_ concentration [23,24,26,30,31,32,35]. These studies estimated 24-hr average PM_2.5_ concentrations attributable to wildfire smoke, and then used these estimates in several ways to test for effects of wildfire-related smoke. In order to distinguish wildfire smoke from background levels, most studies used a wildfire smoke presence interaction term with PM_2.5_ representing pre-, during-, and postwildfire periods. Gan et al. [23] used modeling techniques, in addition to information from the National Oceanographic and Atmospheric Administration’s Hazard Mapping System, to differentiate wildfire smoke-related PM from background PM_2.5_ concentrations. The remaining two studies [25,33] modeled relative risk for wildfire smoke exposed versus unexposed areas or times. 

Studies used a variety of models to estimate wildfire smoke presence and PM concentration. Three studies [23,26,30] used the Weather Research and Forecasting with Chemistry (WRF-Chem) model [36]. Two studies [23,31] used interpolated data from air monitoring stations. Two studies [24,32] used the Hybrid Single Particle Lagrangian Integrated Trajectory (HYSPLIT) Model [37], which uses satellite images, smoke emission estimates, and meteorological measurements to estimate PM_2.5_ concentrations. Two studies [33] used the Goddard Earth Observing System chemical transport model (GEOS-Chem) [38] in combination with monitored data to estimate daily wildfire-specific PM_2.5_ concentration for six years (2004–2009). Rappold et al. [25] used aerosol optical depth (AOD) to determine the presence of the wildfire smoke wave. Gan et al. [23] was the only study to use multiple predicted exposure definitions. In addition to Weather Research and Forecasting Model (WRF)-Chem method, they applied kriging and geographically weighted ridge regression (GWR). Because a separate analysis [39] found that kriging and GWR provided better prediction, according to model fit, of surface measurements of PM_2.5_, we used estimates of relative risk based on GWR-modeled exposures from Gan et al. [23]. 

#### 3.2.1. Health Outcomes

Studies drew health outcome data, shown in Table 1, from hospital and ED admissions records in the study areas. Studies modeled respiratory-related morbidity, measured as hospital admissions [31,33] or ED admissions [24,25,34,35], or both [23,26,30,32]. Liu et al. [33] was the only study to focus solely on the elderly (ages 65+; Medicare recipients).

Table 1 shows that most studies modeled response of a general category of respiratory-related admissions to smoke exposure [23,25,26,30,31,32,33,34,35]. This general category was variously defined, according to comparison of International Classification of Diseases, Ninth (ICD-9) codes, with the most narrow definition by Reid et al. [26], and the broadest definition by Hutchinson et al. [32]. Some studies examined response of specific respiratory conditions, including asthma [23,24,26,30,31,32,34,35]; bronchitis [23,31,32]; chronic obstructive pulmonary disease (COPD) [23,26,31,35]; upper respiratory infection [31,32,35]; pneumonia [23,26,31,32]; and other respiratory infections, symptoms, or diseases [30,34,35]. We report findings for all respiratory conditions, asthma, COPD, bronchitis, and pneumonia only, and do not report other outcomes due to scarce or inconsistently defined categories. 

#### 3.2.2. Covariates

Seven of the ten studies adjusted regression estimates for meteorological factors such as temperature [23,26,30,31,32,33,35], while fewer adjusted for relative humidity [23,26,31,32,35], wind speed and precipitation [23], and surface pressure gradient [31]. Some studies also adjusted for day of the week [23,31,32,35] and year [33]. Delfino et al. [31] also adjusted for fungal spore counts (for asthma) and zip code-level demographic characteristics (age, gender, race, and income). In addition, three studies adjusted for sociodemographic factors such as age, sex, race [33], median income, population over 65 years, housing occupancy status, education, smoking prevalence [26], and percent of the population in poverty [35].

#### 3.2.3. Lags

Each study estimated relative risk for lagged exposure, since they anticipated a delay between exposure, the development of symptoms and admission to an ED or hospital. All studies expected these lags to be relatively short for acute respiratory health outcomes, ranging from 1 to 5 days. As shown in Table 1, five studies tested multiple lags. Studies that tested lags did so for single days and/or, in most cases, for averaging periods (multiple-day lags). 

### 3.3. Stratified Estimates

Selected studies examined a variety of potential modification variables (see Table 1). We report meta-RRR estimates for age and sex categories. Among the 10 studies included in our review, five included RR estimates for all respiratory outcomes and asthma [23,25,26,31,35], four included RR estimates for COPD [23,25,26,35], and three included RR estimates for pneumonia [23,25,26] for both men and women. Eight of the 10 studies included in our review included RR estimates for all respiratory outcomes by age group [23,25,26,30,31,32,34,35]. 

In addition, we report only RRR for study populations stratified by income, race, education, and housing occupancy, without calculation of meta-RRR, because stratified estimates come from single studies, including Reid et al. [26], Liu et al. [33], or Rappold et al. [24]. These studies did not calculate RRRs, and therefore our calculations provide additional between-group comparison for these studies.

Through meta-analysis, we found evidence of higher relative risks for asthma (RRR: 1.038, 95% CI: 1.016, 1.060) in relation to wildfire smoke among women, relative to men (Table 2). The estimate from Reid et al. [26] was influential in the significance of the result; however, with the study removed meta-RRR is 1.033 (95% CI: 0.987, 1.082) (Table 3). We also found evidence of higher relative risks for COPD-related admissions (RRR: 1.018, 95% CI: 1.003, 1.032) among women relative to men. However this meta-estimate is highly influenced by the RR reported by Rappold et al. [25], and should be interpreted with caution given that all of the RRs include the value of 1.0. There was no significant difference between RR among women versus men for pneumonia. Because all *I*^2^ are below 60%, there is little evidence that these meta-estimates are biased by unmodeled heterogeneity [40], although it must be noted that *I*^2^ for asthma was almost 40%, as opposed to <0.0% for other outcomes.

There was evidence of lower relative risk for all respiratory-related hospital or ED admissions in relation to wildfire smoke for youth relative to adults (RRR: 0.986, 95% CI: 0.979, 0.993; *I*^2^: 47.7%) (Table 4). There seems to be more residual heterogeneity in these estimates than in stratification by sex.

Reid et al. [26], Liu et al. [33], and Rappold et al. [24] were the only studies to report relative risk stratified by other demographic characteristics. The RRRs for each strata are shown in Table 5, and provide between-group comparison not provided in original studies. However, because groups were defined using different terms, and at most two studies provide estimates for only one stratification category, we did not calculate meta-RRRs for these strata.

Reid et al. [26] estimated associations with multiple health outcomes including all respiratory, asthma, COPD and pneumonia using hospital and ED admissions within zip codes affected by smoke in the northern California study area from wildfires that occurred in 2008. They evaluated effect modification by tertiles of household median income, percent of the population living in owner-occupied housing, percent of the population with less than a high school diploma, and percent white, with data obtained for each zip code from the US Census (Table 5). They found evidence of heterogeneity in the effect of wildfire smoke exposure, with higher relative risks (RR) observed for lowest vs. highest tertile income for all respiratory-related admissions, mid- vs. lowest tertile educational attainment for pneumonia-related admissions, and highest vs. lowest tertile educational attainment for COPD-related admissions.

Liu et al. [33] estimated the association between respiratory hospital admissions among Medicare recipients (age 65+) within 561 counties of the US on smoke-wave days compared to non-smoke-wave days. They examined effect modification by educational attainment (+/− 20% of the population with a high school diploma), percent poverty (<10%, 10–15%, and >15%), and by race (White, Black and other). Table 5 shows that RRR calculations from this study do not indicate a measurable difference in risk by education or between racial groups, however there is an increased risk for low (<10%) versus medium poverty (10–15%) groups (RRR: 1.160, 95% CI: 1.000, 1.347). Liu et al. [33] also examined effect modification by urbanity (urban versus less urban; Table 6) and by region of the US (California, Northwest, Southwest, and Rocky Mountains) for the effect of wildfire smoke. RRR calculations indicate no measurable differences in risk by either level of urbanity or region.

Rappold et al. [24] estimated cumulative relative risk for asthma-related ED visits during 3-day dense smoke exposure times in counties exposed to peat bog wildfire smoke versus in unexposed counties (not per increase in PM_2.5_ exposure). They examined a variety of stratification variables, categorized as health behaviors (including indicators of tobacco smoking, diet and exercise, alcohol use, and unsafe sex), clinical care (including indicators of access to care and quality of care), socioeconomic factors (including indicators of education, employment, income, family and social support, and community safety), and the physical environment (including indicators of environmental quality and the built environment). They generated estimates of cumulative relative risk for groups above and below median values within each category, with above-median groups representing more desirable outcomes. While they found that below-median socioeconomic status and health behaviors indicated a higher RRR, confidence intervals for all RRRs calculated included the value of 1.0 (Table 5 and Table 6).

## 4. Discussion

We examined evidence of heterogeneity in the effect of wildfire smoke on respiratory health in North America and report evidence of effect modification by sex and age. Namely, we found higher relative risk for females than for males, for asthma, and for COPD, and for adults than for youth for all respiratory-related hospital or ED admissions. We reported evidence of heterogeneity of effect from selected studies by other categories, though based on single studies, and found some evidence of higher relative risk for low versus high income groups, for all respiratory admissions. We also found lower relative risk of pneumonia-related admissions for low versus middle educational attainment groups, and of COPD admissions for low versus high educational attainment groups. While risk estimates could be attributed to confounding, measurement error, and other sources of bias, our calculations of heterogeneity, especially for sex, and the use of the ratio of relative risk mitigate these concerns.

The mechanisms of these heterogeneous effects are unclear. Because respiratory systems of youth are developing, we would expect that youth would have different level of risk than adults for all respiratory-related hospital or ED admissions associated with wildfire smoke exposure. Women may be more susceptible to airway restriction that occurs with asthma due to relatively smaller respiratory airways, however this does not explain relative susceptibility to COPD which is typically a result of long-term damage, e.g., from smoking. 

Meta-estimates of RRR serve as general indicators of differences in association between wildfire smoke exposure and hospital or ED admissions for respiratory outcomes. While RRR can be used to assess for heterogeneity of effect, estimates of RR should not be compared directly because studies differ greatly in methods for exposure measurement, definition of case vs. referent categories, health outcome definitions, ecological and geographic settings, lag periods, and other modeling specifications. 

Many gaps remain in evidence necessary to inform public policy and forest management strategy. First, our calculations of RRR for most stratification variables are based on single studies. Because all studies pertain to single or statewide wildfire complexes, and populations are local, estimates may not apply outside the study area and population. 

Where more than one study produced estimates for modification by group characteristics, studies often employed different stratification group definitions. In other words, pooling of effect estimates was not possible, or in some cases was forced on the data in the presence of heterogeneity of groups being pooled, which could bias RRR values. For example, we included estimates derived from populations of varying ages in each of our age categories. While RR estimates for the elderly were standardly defined (ages 65+), RR estimates for youth were based on data from participants of lowest age ranging from 0 to 5, and highest age ranging from 14 to 19. RR estimates for adults were based on data from participants of lowest age ranging from 15 to 20, and highest age of 64. Meta-RRR estimates by respiratory outcome, comparing females to males, were based on RR estimates based on data from participants of varying age ranges (e.g., all ages, elderly, and adults for all respiratory symptoms). 

Lack of consistency in defining and measuring exposure hampered our analysis. Only one study included in our review compared risk estimates based on multiple exposure models [23], and found that risk estimates varied, enough to alter interpretations, based on exposure measurement technique. In some cases, our heterogeneous RRR calculations could be due to this bias alone.

Another limitation of our analysis and its wider application is that the nature of exposure may vary due to local factors. Chemical composition of wildfire smoke, and thereby toxicity, can also vary by region, climate, season, burn condition, stage of wildfire (open flames versus smoldering), and vegetation (fuel) type [11]. It should be noted that all risk estimates from the eastern US are based on smoke exposure from peat fires which may have a different chemical composition and, thereby, affect various subpopulation groups differently than smoke from forest fires. In addition, underlying health conditions of populations could vary by study context, and could influence rates of hospital and ED admissions.

## 5. Conclusions

Estimation of heterogeneity of effect of wildfire smoke on population subgroups is an emerging area of study. We provide evidence of heterogeneity in the effects of wildfire smoke exposure on respiratory health in North American setting. More work to understand the magnitude of differential risk among population subgroups is necessary in order to develop appropriate public health measures, such as messaging, in an attempt to reduce risk among more vulnerable populations.

## Figures and Tables

**Table 1:** Study Characteristics.

	Citation	Location	Study Period	Wildfire Event	Study Design	Age	Visit Types	Health Outcomes	ICD-9 Codes	Effect Modifiers	Referent	Lags	Exposure Contrast
1	Alman et al. (2016) [30]	Colorado	26 March–10 July 2012	Statewide forest wildfire complex	Case crossover	all ages	ED and hospital admissions	All respiratory conditions, asthma, bronchitis, COPD, pneumonia, upper respiratory infection	460–66, 480–88, 490–92, 496, 493–786.07, 460–5, 466.0, 466.1, 466.11, 466.19	(1) age (0–18, 19–64, 65+)	grid-specific nonexposure	0, 0–1, 0–1–2	5 µg/m^3^
2	Delfino 2008 [31]	Southern California	2003	Region-wide forest wildfire complex	Time series	all ages	Hospital admissions	All respiratory conditions, asthma, bronchitis & bronchiolitis, COPD, pneumonia, upper respiratory infection	493, 466, 491–92, 496, 480–87, 460–65	(1) age (0–4, 5–19, 20–64, 65–99; COPD: 20–64, 65–99); (2) sex [not for all categories]	Prewildfire (compared to during and post)	0–1	10 µg/m^3^
3	Gan et al. (2017) [23]	WA State	1 July–31 October 2012	Statewide forest wildfire complex	"Time-stratified case crossover"	all ages	ED and hospital admissions	All respiratory conditions, asthma, bronchitis, COPD, pneumonia	460–519, 480–86, 466	(1) age (<15, 15+, 15–65); (2) sex	Subject-specific nonexposure	none, 0, 0–1, 0–2, 0–3, 0–4, 0–5	10 µg/m^3^
4	Hutchinson et al. (2018) [32]	Southern California	22 October–5 November 2007	Region-wide forest wildfire complex	Case crossover	all ages	ED and hospital admissions	All respiratory conditions, asthma, bronchitis, pneumonia, upper respiratory infection	277, 460–64, 466, 480–87, 490–96, 506, 508, 786	(1) age (0–1, 2–4, 0–4, 5–17, 18–64)	Non-smoke exposed times; subject-specific nonexposure	none	10 µg/m^3^
5	Liu et al. (2017a) [33]	Western USA	2004–2009	Region-wide forest wildfire complex	Ecological	65+	Hospital admissions	All respiratory conditions	490–492, 464–466, 480–487	(1) age (65+, 65–74, 85+), (2) sex, (3) region (urban, rural, CA, NW, SW, Rocky Mountain), (4) education (>20%, <20% with bachelor degree), race (white, black, other), (5) poverty (<10%, 10–15%, 15%+)	Non-smoke exposed times	none	none
6	Rappold et al. (2011) [25]	North Carolina	1 June–14 July 2008	Peat wildfire in Pocosin Lakes National Wildlife Refuge	Ecological	all ages	ED admissions	All respiratory conditions	465, 466, 480, 481, 482, 483, 484, 485, 486, 490, 491, 492, 493	(1) age (all, 65+, <65); (2) sex; (3) region	Non-smoke exposed areas	0–5	none
7	Rappold et al. (2012) [24]	North Carolina	1 June–14 July 2008	Peat wildfire in Pocosin Lakes National Wildlife Refuge	Time series	18+	ED admissions	Asthma	428, 493	(1) health behaviors (tobacco use, diet/exercise, alcohol use, unsafe sex); (2) clinical care (access to care, quality of care); (3) SES (education, employment, income, family & social support, community safety); (4) physical environment (environmental quality, built environment)	Non-smoke exposed areas	0, 1, 0–1	100 µg/m^3^
8	Reid et al. (2016) [26]	Northern California	2008	Large wildfire complex	Time series	all ages	ED and hospital admissions	All respiratory conditions, asthma, COPD, pneumonia	493	(1) age (<20, 20–64, 65+); (2) sex; (3) race; (4) median income; (5) percent of the population with less than a high school diploma; (6) percent of owner–occupied housing units	Prewildfire (compared to during and post)	0–1,0–2	5 µg/m^3^
9	Resnick et al. (2013) [34]	Albuquerque, New Mexico	2011	Wallow Fire forest wildfire	Time series	all ages	ED admissions	All respiratory conditions, asthma, other diseases of respiratory system	460–519	(1) age (0–19, 20–64, 73+)	Prewildfire (compared to during and post)	none	10 µg/m^3^
10	Tinling et al. (2016) [35]	North Carolina	5 May 5–15 June 2011	Pains Bay peat wildfire	Time series	all ages	ED admissions	All respiratory conditions, asthma, COPD, upper respiratory infection, other chest/respiratory symptoms	786	(1) age (<18, 18–64, 18+, 65+); (2) sex	Non-smoke exposed times	0–2	10 µg/m^3^

ICD: International Classification of Disease; ED: Emergency Department; COPD: Chronic Obstructive Pulmonary Disease; SES: Socioeconomic Status.

**Table 2:** Ratio of relative risks for estimates of respiratory outcomes associated with wildfire smoke exposure, stratified by sex.

Study	Visit Type	Age	Lag	Female:Male		
				RR	95% CI	*I* ^2^
All Respiratory						
Gan et al. 2017	ED	Adult (15–64)	none	0.998	(0.947, 1.052)	
Liu et al. 2017	ED	Elderly (65+)	none	0.929	(0.786, 1.097)	
Rappold et al. 2011	ED	Ages 19+	0–5	1.124	(0.764, 1.654)	
Reid et al. 2016	ED	All ages	0–2	1.018	(0.995, 1.041)	
Tinling et al. 2016	ED	Adult (18–64)	0–2	1.029	(0.939, 1.128)	
	Meta-RRR:			1.015	(0.994, 1.035)	0.0%
Asthma						
Delfino et al. 2008	Hospital	Adult (20–64)	none	1.056	(1.000, 1.114)	
Gan et al. 2017	ED	Adult (15–64)	none	0.998	(0.895, 1.113)	
Rappold et al. 2011	ED	Ages 19+	0–5	1.900	(0.941, 3.838)	
Reid et al. 2016	ED	All	0–2	1.039	(1.014, 1.065)	
Tinling et al. 2016	ED	Adult (18–64)	0–2	0.919	(0.796, 1.060)	
	Meta-RRR:			1.038	(1.016, 1.060)	38.5%
COPD						
Gan et al. 2017	ED	Adult	none	1.075	(0.954, 1.212)	
Rappold et al. 2011	ED	All	0–5	0.594	(0.214, 1.648)	
Reid et al. 2016	ED	All	0–2	1.017	(0.992, 1.023)	
Tinling et al. 2016	ED	Adult	0–2	1.017	(0.981, 1.055)	
	Meta-RRR:			1.018	(1.003, 1.032)	0.0%
Pneumonia						
Gan et al. 2017	ED	Adult	none	0.987	(0.892, 1.091)	
Rappold et al. 2011	ED	All	0–5	0.877	(0.468, 1.645)	
Reid et al. 2016	ED	All	0–2	1.005	(0.979, 1.032)	
	Meta-RRR:			1.004	(0.978, 1.030)	0.0%

RRR: Ratio of Relative Risk.

**Table 3:** Meta-analyses of ratio of relative risks for estimates of respiratory outcomes associated with wildfire smoke exposure stratified by sex, with each study removed.

Study	Visit Type	Age	Lag	Meta-RRR with Each Study Removed ^a^		
				Meta-RRR	95% CI	*I* ^2^
All Respiratory						
Gan et al. 2017	ED	Adult	none	1.017	(0.995, 1.040)	0.0%
Liu et al. 2017	ED	Elderly	none	1.016	(0.995, 1.037)	0.0%
Rappold et al. 2011	ED	All	0–5	1.014	(0.004, 1.035)	0.0%
Reid et al. 2016	ED	All	0–2	1.002	(0.959, 1.046)	0.0%
Tinling et al. 2016	ED	Adult	0–2	1.014	(0.993, 1.035)	0.0%
Whole Group Meta-RRR:				1.015	(0.994, 1.035)	0.0%
Asthma						
Delfino et al. 2008	Hospital	Adult	none	1.034	(1.010, 1.059)	50.3%
Gan et al. 2017	ED	Adult	none	1.039	(1.017, 1.063)	49.9%
Rappold et al. 2011	ED	All	0–5	1.037	(1.015, 1.060)	18.0%
Reid et al. 2016	ED	All	0–2	1.033	(0.987, 1.082)	53.6%
Tinling et al. 2016	ED	Adult	0–2	1.041	(1.018, 1.064)	100.0%
Whole Group Meta-RRR:				1.038	(1.016, 1.060)	38.5%
COPD						
Gan et al. 2017	ED	Adult	none	1.017	(1.003, 1.031)	0.0%
Rappold et al. 2011	ED	All	0–5	1.018	(1.004, 1.032)	0.0%
Reid et al. 2016	ED	All	0–2	1.021	(0.986, 1.057)	0.0%
Tinling et al. 2016	ED	Adult	0–2	1.018	(1.002, 1.033)	0.0%
Whole Group Meta-RRR:				1.018	(1.003, 1.032)	0.0%
Pneumonia						
Gan et al. 2017	ED	Adult	none	1.005	(0.979, 1.032)	0.0%
Rappold et al. 2011	ED	All	0–5	1.004	(0.979, 1.030)	0.0%
Reid et al. 2016	ED	All	0–2	0.984	(0.891, 1.087)	0.0%
Whole Group Meta-RRR:				1.004	(0.978, 1.030)	0.0%

^a^ For example, the meta-RRR listed under all-respiratory health for Gan et al. [23] is the meta-RRR including RRR values from all studies except for Gan et al. [23].

**Table 4:** Ratio of relative risks for estimates of all respiratory outcomes associated with wildfire smoke exposure, stratified by age.

Study	Visit Type	Lag	Ratio of Relative Risks (RRR)					
			Youth:Adult	95% CI	Youth:Elderly	95% CI	Elderly:Adult	95% CI
Alman et al. 2016	ED	0	0.966	(0.938, 0.995)	0.974	(0.930, 1.021)	1.009	(0.967, 1.052)
Delfino et al. 2008	Hospital	0	1.003	(0.955, 1.053)	0.997	(0.950, 1.046)	0.994	(0.968, 1.021)
Gan et al. 2017	ED	0	1.034	(0.954, 1.120)	1.011	(0.938, 1.091)	0.978	(0.923, 1.037)
Hutchinson et al. 2018	ED	0–5	0.983	(0.638, 1.515)				
Rappold et al. 2011	ED	0–5					1.050	(0.874, 1.260)
Reid et al. 2016	ED	0–2	0.969	(0.952, 0.985)	0.977	(0.959, 0.995)	1.009	(0.992, 1.025)
Resnick et al. 2015	ED	0	0.805	(0.667, 0.970)	0.693	(0.537, 0.894)	0.861	(0.680, 1.091)
Tinling et al. 2016	ED	0–2	1.012	(0.972, 1.054)	1.066	(1.017, 1.118)	1.054	(1.010, 1.099)
Meta RRR:			0.976	(0.963, 0.989)	0.987	(0.973, 1.002)	1.008	(0.996, 1.020)
*I*^2^:				47.7%		74.6%		27.0%

**Table 5:** Ratio of relative risks for stratified estimates of all respiratory outcomes associated with wildfire smoke exposure, stratified by socioeconomic factors.

Study	Health Outcome	Ratio of Relative Risks (RRR)					
		Low:Middle	95% CI	Low:High	95% CI	Middle:High	95% CI
Income ^a^							
Reid et al. 2016	All respiratory	1.009	(0.994, 1.022)	1.019	(1.004, 1.033)	1.010	(0.996, 1.024)
Reid et al. 2016	Asthma	1.012	(0.984, 1.039)	1.021	(0.990, 1.051)	1.009	(0.978, 1.040)
Reid et al. 2016	COPD	1.020	(0.982, 1.059)	1.039	(0.997, 1.082)	1.018	(0.976, 1.061)
Reid et al. 2016	Pneumonia	1.009	(0.977, 1.040)	1.017	(0.981, 1.054)	1.009	(0.977, 1.041)
% Occupied Housing ^b^							
Reid et al. 2016	All respiratory	0.999	(0.986, 1.012)	0.998	(0.982, 1.014)	0.999	(0.983, 1.015)
Reid et al. 2016	Asthma	0.989	(0.962, 1.014)	0.998	(0.966, 1.031)	1.010	(0.978, 1.042)
Reid et al. 2016	COPD	1.008	(0.968, 1.048)	1.036	(0.985, 1.088)	1.028	(0.982, 1.075)
Reid et al. 2016	Pneumonia	1.002	(0.971, 1.033)	1.004	(0.969, 1.040)	1.003	(0.969, 1.036)
% Population with High School Diploma ^c^							
Liu et al. 2017	All respiratory			0.988	(0.918, 1.062)		
Reid et al. 2016	All respiratory	0.990	(0.974, 1.004)	0.991	(0.974, 1.006)	1.001	(0.987, 1.014)
Reid et al. 2016	Asthma	0.994	(0.963, 1.025)	0.996	(0.965, 1.027)	1.002	(0.974, 1.029)
Reid et al. 2016	COPD	0.982	(0.943, 1.022)	0.959	(0.920, 0.997)	0.976	(0.939, 1.013)
Reid et al. 2016	Pneumonia	0.967	(0.935, 0.999)	0.990	(0.956, 1.025)	1.024	(0.993, 1.056)
% Poverty ^d^							
Liu et al. 2017	All respiratory	0.862	(0.720, 1.032)	1.160	(1.000, 1.347)	1.000	(0.862, 1.161)
Socioeconomic factors ^e^							
Rappold et al. 2012	All respiratory			1.113	(1.000, 1.347)		
Race ^f^							
Reid et al. 2016	All respiratory	0.992	(0.979, 1.005)	0.992	(0.976, 1.008)	1.000	(0.984, 1.015)
Reid et al. 2016	Asthma	0.986	(0.959, 1.013)	1.002	(0.968, 1.038)	1.017	(0.983, 1.051)
Reid et al. 2016	COPD	0.992	(0.955, 1.030)	1.008	(0.965, 1.051)	1.015	(0.974, 1.057)
Reid et al. 2016	Pneumonia	0.978	(0.946, 1.009)	0.997	(0.962, 1.033)	1.020	(0.987, 1.053)
		**White:Black**	**95% CI**	**White:Other**	**95% CI**	**Black:Other**	**95% CI**
Liu et al. 2017	All respiratory	0.877	(0.714, 1.076)	1.029	(0.915, 1.155)	1.173	(0.947, 1.452)

Notes: ^a^ ED admissions; 0–2 Lag; Groups represent tertiles. ^b^ ED admissions; 0–2 Lag; Groups represent tertiles. ^c^ Reid et al. 2016: ED admissions, 0–2 Lag, groups represent tertiles; Liu et al. 2017: ED & hospital admissions, 0 Lag; categories defined as <20%: >20% high school diploma. ^d^ Hospital admissions; Poverty categories defined as low: <10%, medium: 10–15%, and high: >15%. ^e^ RRR estimates represent below: above median values. ^f^ Reid et al. 2016: ED admissions; 0–2 Lag, Categories defined as tertile of % White.

**Table 6:** Ratio of relative risks for estimates of all respiratory outcomes associated with wildfire smoke exposure, stratified by health behaviors, access to care, and physical environment.

Stratification Variables	Ratio of Relative Risks	95% CI
Rappold et al. 2012 ^a^		
Health Behaviors	1.631	(0.966, 2.752)
Access to Care	0.822	(0.486, 1.391)
Physical Environment	0.712	(0.405, 1.251)
Liu et al. 2017		
Urban: Less Urban	0.955	(0.810, 1.127)
CA: Northwest	0.813	(0.616, 1.072)
CA: Southwest	0.954	(0.440, 2.069)
CA: Rocky Mountains	1.000	(0.699, 1.431)
Northwest: Southwest	1.174	(0.520, 2.653)
Northwest: Rocky Mountains	1.231	(0.793, 1.911)
Southwest: Rocky Mountains	1.048	(0.450, 2.442)

^a^ RRR estimates represent below: above median values.
